# Nurse Practitioner–Led Integrated Rapid Access to HIV Prevention for People Who Inject Drugs (iRaPID): Protocol for a Pilot Randomized Controlled Trial

**DOI:** 10.2196/42585

**Published:** 2022-10-11

**Authors:** Antoine Khati, Frederick L Altice, David Vlahov, William H Eger, Jessica Lee, Terry Bohonnon, Jeffrey A Wickersham, Francesca Maviglia, Nicholas Copenhaver, Roman Shrestha

**Affiliations:** 1 Department of Allied Health Sciences University of Connecticut Storrs, CT United States; 2 AIDS Program Yale School of Medicine New Haven, CT United States; 3 Yale School of Nursing West Haven, CT United States

**Keywords:** HIV prevention, people who inject drugs, sexual risk, pre-exposure prophylaxis, opioid agonist therapy, medications for opioid use disorder, opioid use disorder

## Abstract

**Background:**

The ongoing volatile opioid epidemic remains a significant public health concern, alongside continued outbreaks of HIV and hepatitis C virus among people who inject drugs. The limited access to and scale-up of medications for opioid use disorder (MOUD) among people who inject drugs, coupled with multilevel barriers to pre-exposure prophylaxis (PrEP) uptake, makes it imperative to integrate evidence-based risk reduction and HIV prevention strategies in innovative ways. To address this need, we developed an integrated rapid access to HIV prevention program for people who inject drugs (iRaPID) that incorporates same-day PrEP and MOUD for this population.

**Objective:**

The primary objective of this pilot study is to assess the feasibility and acceptability of the program and evaluate its preliminary efficacy on PrEP and MOUD uptake for a future randomized controlled trial (RCT). We also aim to explore information on the implementation of the program in a real-world setting using a type I hybrid implementation trial design.

**Methods:**

Using a type I hybrid implementation trial design, we are pilot testing the nurse practitioner–led iRaPID program while exploring information on its implementation in a real-world setting. Specifically, we will assess the feasibility and acceptability of the iRaPID program and evaluate its preliminary efficacy on PrEP and MOUD uptake in a pilot RCT. The enrolled 50 people who inject drugs will be randomized (1:1) to either iRaPID or treatment as usual (TAU). Behavioral assessments will occur at baseline, and at 1, 3, and 6 months. Additionally, we will conduct a process evaluation of the delivery and implementation of the iRaPID program to collect information for future implementation.

**Results:**

Recruitment began in July 2021 and was completed in August 2022. Data collection is planned through February 2023. The Institutional Review Boards at Yale University and the University of Connecticut approved this study (2000028740).

**Conclusions:**

This prospective pilot study will test a nurse practitioner–led, integrated HIV prevention program that incorporates same-day PrEP and MOUD for people who inject drugs. This low-threshold protocol delivers integrated prevention via one-stop shopping under the direction of nurse practitioners. iRaPID seeks to overcome barriers to delayed PrEP and MOUD initiation, which is crucial for people who inject drugs who have had minimal access to evidence-based prevention.

**Trial Registration:**

ClinicalTrials.gov NCT04531670; https://clinicaltrials.gov/ct2/show/NCT04531670

**International Registered Report Identifier (IRRID):**

DERR1-10.2196/42585

## Introduction

With opioid use disorder being intricately linked to HIV transmission via drug injection, the United States is witnessing resurgent twin opioid and HIV epidemics, fueled by concurrent rises in opioid-related overdoses [1-6]. People who inject drugs remain disproportionately affected by HIV [7] despite their engagement in preventable drug- and sex-related risk behaviors [8-14]. There is an urgent need to combine evidence-based harm reduction services and HIV prevention strategies in innovative ways to effectively curb HIV incidence.

Critical elements in the HIV prevention toolbox for people who inject drugs include medication for opioid use disorder (MOUD), syringe services programs (SSPs), pre-exposure prophylaxis (PrEP), and HIV treatment as prevention. MOUD minimizes the risk of fatal overdose and effectively reduces HIV transmission by decreasing or eliminating the frequency of injection [15-21], while SSPs narrow transmission risk with each protected injection [22,23]. Nevertheless, access to these programs among people who inject drugs is often limited, with overall coverage remaining low [24-27] despite data documenting increased HIV incidence in people who inject drugs where MOUD and SSPs are unavailable [28-30]. HIV treatment as prevention requires active case-finding and effective linkage to HIV treatment, which is markedly improved when people with HIV gain access to MOUD [31].

PrEP is an essential additional tool for people who inject drugs [32,33]. It is not restricted to specially licensed prescribers and reduces HIV transmission from not only drug injection but also sex. Although integrating PrEP into existing evidence-based risk reduction programs can strengthen HIV prevention efforts for people who inject drugs [16,17,19-21,34], its scale-up has been hampered for several reasons. First, PrEP and MOUD each require a separate prescription. They are often delivered in different settings and by clinicians with varying areas of expertise [35], generating excess demands on patients and making access to care more difficult for people who inject drugs. Furthermore, the provision of PrEP and MOUD prescriptions is unnecessarily complex. The traditional PrEP delivery model requires multiple visits before the prescription. Existing data show high attrition rates between initial screening and PrEP initiation, adding to the attrition observed between PrEP referral and initiation [36,37]. The prescription of MOUD also has its own set of demands [38], some of which are regulated by the Drug Enforcement Agency. PrEP and MOUD have been mainly prescribed in a “centralized” care delivery model that focuses more on physicians, is costly, and limits accessibility. There also remains a suboptimal supply of prescribers [39-41], with many physicians reluctant to prescribe medications to people who inject drugs [42-45].

An innovative approach of coprescribing PrEP and MOUD on the same day can streamline delivery, as it addresses impediments to PrEP and MOUD uptake. Same-day PrEP prescription is a safe, feasible, and acceptable approach to linking at-risk individuals to much-needed PrEP care early on [46-48] while decreasing attrition rates between the initial visit and prescription time and increasing adherence [49]. This approach also sets the stage to boost PrEP uptake and makes the current standard of care, which already comprises streamlined access to and initiation of MOUD, more inclusive. The provision of same-day MOUD, when integrated with PrEP, provides the added benefit of stabilizing patients and can therefore strongly complement and facilitate PrEP initiation, adherence, and persistence as has been observed with antiretroviral therapy [34,50]. Additionally, it has the potential to overcome provider-level barriers, increasing the confidence that patients will maintain their treatment regimen [43,45].

With ongoing calls for task shifting to support the expansion of HIV prevention services [51], there is a need to “decentralize” PrEP delivery by incorporating it into front-line prevention services provided by nurse practitioners (NPs). Nurse-led PrEP delivery is a viable strategy that can lead to rapid increases in PrEP service capacity without additional resources [39,52]. Furthermore, the 2016 Comprehensive Addiction and Recovery Act permits NPs to prescribe buprenorphine with waivers, paving the way for PrEP and MOUD coprescription. This decentralized delivery model represents an innovative approach to scale-up integrated HIV prevention services among people who inject drugs.

We developed an NP-led, integrated rapid access to HIV prevention program for people who inject drugs (iRaPID) that incorporates same-day access to PrEP and MOUD. The primary objective of this pilot study is to assess the feasibility and acceptability of the program and evaluate its preliminary efficacy on PrEP and MOUD uptake for a future randomized controlled trial (RCT). We also aim to explore information on the implementation of the program in a real-world setting using a type I hybrid implementation trial design.

## Methods

### Study Design

The protocol involves a type I hybrid implementation trial design [53], where we pilot test the NP-led iRaPID program. The study is a 2-arm RCT that examines the feasibility and acceptability of the program among people who inject drugs and clinical stakeholders. In the process, we can provide a preliminary estimate of the efficacy of PrEP and MOUD uptake by comparing the iRaPID program against treatment as usual (TAU) among HIV-negative people who inject drugs. We hypothesize that the iRaPID program will be more efficacious than will TAU on PrEP and MOUD uptake and adherence.

In this type I hybrid implementation trial design, we collect process measures to inform future implementation efforts. We will explore potential patient- and provider-level barriers and facilitators of the program regarding its implementation in real-world settings. More specifically, we will assess at both these levels the overall satisfaction, perceived utility, and relevance of each intervention component; determine the relevant needs of the target population (ie, people who inject drugs), thereby delineating intervention characteristics that address those needs; and explore the barriers to implementation of the iRaPID program, such as logistical barriers and time and resource constraints. Finally, changes needed to accommodate same-day coprescription of PrEP and MOUD will be established, along with the resources and structural changes necessary for the successful implementation of the program.

### Ethics Approval

The institutional review board at Yale University approved this protocol (#2000028740) with an institutional reliance agreement with the University of Connecticut. This study is registered at ClinicalTrials.gov (NCT04531670).

### Study Setting

This study is being conducted at the New Haven Syringe Service Program (NHSSP) in Connecticut, the first SSP in the United States (established 1986). With steadily high-ranking and rising fatal and nonfatal accidental overdose rates over the past few years, New Haven continues to bear the detrimental impacts of a severe opioid crisis and reflects the nationwide picture of a twin opioid and HIV epidemic, with a high HIV prevalence among people who inject drugs in the city [54,55]. The NHSSP offers services beyond New Haven to exurban and rural communities and integrated medical services through the Community Health Care Van, a 40-foot mobile medical clinic, and minivans that provide personalized services [56-60].

### Study Procedures

#### Recruitment and Screening

Recruitment for the trial started in July 2021 at the NHSSP and lasted until August 31, 2022. People who inject drugs were recruited on-site through posted flyers at the central and mobile distribution locations of the NHSSP, word of mouth, and community-based outreach. Individuals are screened initially by phone or in person to assess whether they meet the eligibility criteria, which include being 18 years or older; having a confirmed HIV-negative status; self-reported injection drug use in the past 6 months; OUD based on *The Diagnostic and Statistical Manual of Mental Disorders, Fifth Edition* criteria; and substantial ongoing HIV risk factors (eg, condomless sex, needle sharing) in the past 6 months. Uninsured participants were referred to an on-site case manager for assistance with insurance enrollment.

#### Informed Consent, Enrollment, and Randomization

After confirming eligibility criteria and providing informed consent, all participants underwent a baseline assessment, which took approximately 90 minutes and was administered electronically by a trained research staff member in a private room at NHSSP. Participants were then randomized (1:1) to receive either the iRaPID intervention or TAU. Randomization was done via REDCap software (Vanderbilt University), a secure, HIPAA (Health Insurance Portability and Accountability Act)-compliant web app with a built-in randomization feature that allows the development of a defined randomization model. Randomization was stratified by sex to ensure that an equal proportion of participants is present in both arms. Participants are assessed at baseline, and 1-, 3-, and 6-month time points. This approach allows us to examine short-term outcomes and the decay and emergence of intervention effects. Study participants are reimbursed for the time required to complete the assessments (US $35 for the baseline assessment and US $25 for each follow-up assessment).

### Intervention Procedures

#### Treatment as Usual

Participants randomized to the TAU group will follow the existing clinical guidelines to receive PrEP, MOUD, or both medications. They are provided with a comprehensive list of PrEP and MOUD programs available in the area. Since PrEP and MOUD services are not integrated within the current standard of care, participants will have to be screened at separate visits (one for PrEP and another for MOUD) if they are interested in both programs unless they find a prescriber who will provide both.

**Figure 1:**
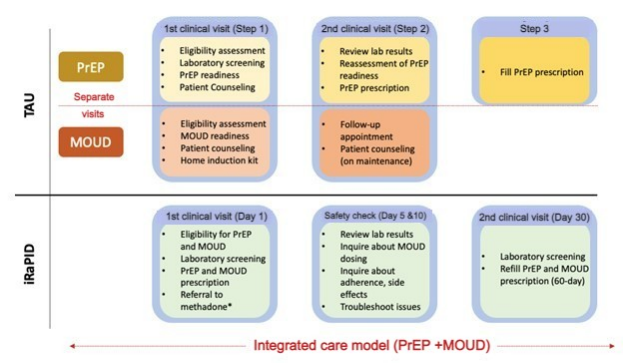
Integrated care process for same-day PrEP and MOUD compared to TAU. iRaPID: integrated rapid access to HIV prevention program for people who inject drugs; MOUD: medication for opioid use disorder; PrEP: pre-exposure prophylaxis; TAU: treatment as usual. *Patients interested in methadone prescriptions are referred to a methadone (or other appropriate) addiction treatment center.

For PrEP, the current standard of care generally involves 2 clinical appointments. During the first visit, patients are assessed for eligibility and PrEP readiness and receive additional counseling on using PrEP. Additionally, laboratory screenings are ordered. During the second visit, which usually occurs 1 to 3 weeks later, the laboratory results are reviewed, PrEP readiness is reassessed, and the patient is provided with a PrEP prescription that they will need to fill at a pharmacy. Access to MOUD also entails 2 separate clinic visits at least 3 days apart. The first encompasses an eligibility assessment, patient counseling, and an evaluation of the patient's readiness to start therapy. Patients are also given a home induction kit and asked to follow up in 3 days for further counseling on maintenance therapy (Figure 1).

#### iRaPID Intervention

The iRaPID program was developed by adapting TAU services for PrEP and MOUD to accommodate same-day coprescription for both medications through a single nurse provider. We used the modified participatory, evidence-based, patient-focused process for advanced practice nursing [61] to guide the optimal use of the NP-led model and refined it to incorporate the same-day coprescription of PrEP and MOUD. Participants randomized to the iRaPID group will receive a prescription for PrEP and MOUD and educational counseling by an NP, followed by safety checks via phone calls or SMS text messages on days 5 and 10; and a clinical visit on day 30 (Figure 1).

During the first day, the NP evaluates the participant's eligibility for PrEP and assesses PrEP readiness. Participants eligible for PrEP are evaluated using the Centers for Disease Control and Prevention's PrEP clinical practice guidelines [62], including acute HIV infection symptoms and laboratory testing for HIV, hepatitis (ie, hepatitis A, B, and C viruses), creatinine, pregnancy (for females), and sexually transmitted infections (ie, gonorrhea, chlamydia, and syphilis). During the same visit, they will also be offered a PrEP prescription for a 30-day supply.

Additionally, the NP assesses MOUD readiness. Participants are screened for buprenorphine maintenance therapy (BMT) eligibility according to the Drug Addiction Treatment Act of 2000 and Treatment Improvement Protocol Series 43 criteria [63]. As methadone maintenance therapy (MMT) is only provided in specialty settings, only BMT is offered at the study site (NHSSP). Patients preferring MMT over BMT are referred to initiate same-day MMT. If the participant is eligible for BMT, a drug toxicology screen is performed, and participants are provided with office or home induction and resources to assist them in self-managing their opioid treatment [64]. The resources provided include a home induction kit with an instruction sheet, BMT film packets, nonsteroidal anti-inflammatory drugs (for body pain), hydroxyzine (for anxiety and insomnia), and loperamide (for diarrhea). Additionally, patients receive adherence and potential medical adverse events counseling.

After the laboratory results become available (typically within 3 days following the visit), the NP contacts the participant (on day 5) via a phone call or SMS text message to review the test results and inquire about MOUD stabilization. If test results are concerning and warrant PrEP discontinuation (which is typically rare) [65-68], participants are urged to cease PrEP immediately. On day 10, the NP contacts the participant using the same modality to inquire about PrEP adherence, possible side effects, and MOUD dosing as well as to troubleshoot potential billing issues. On day 30, the NP prescribes a 60-day supply of PrEP and a 30-day supply of MOUD and provides counseling related to long-term maintenance of PrEP and MOUD. In the case of a positive test result for any disease or laboratory value outside the normal range, participants are referred to appropriate clinical care according to the standard of care at any time point during the study. The study’s timeline and activities are summarized in Table 1.

**Table 1:** Study activity and measures.

Study activity			Timeline													
			Screening (prebaseline)		Evaluation (baseline)		Treatment					Retention				
							Day 5		Day 10		1 month			3 months		6 months
Eligibility assessment			✓													
Informed consent					✓											
**TAU^a^ PrEP^b^**																
	PrEP educational counseling^c^			✓									✓		✓	
	Lab screenings			✓									✓		✓	
	Lab result review^c^			✓									✓		✓	
	PrEP prescription^c^			✓									✓		✓	
**TAU MOUD^d^**																
	Office/home induction			✓												
	Buprenorphine kit			✓												
	Follow-up appointment							✓					✓		✓	
**iRaPID^e^**																
	Educational counseling^f^			✓				✓								
	PrEP prescription			✓						✓			✓		✓	
	Safety check					✓		✓								
	Day 30 check-up			✓												
	Follow-up appointment							✓					✓		✓	
Research interview					✓									✓		✓
DBS^g^											✓			✓		✓
Qualitative interview														✓		✓
Payment					✓						✓			✓		✓

^a^ TAU: treatment as usual.

^b^PrEP: pre-exposure prophylaxis.

^c^Exact day depends on when the laboratory results are available for the clinician (usually 3-7 days after baseline assessment).

^d^MOUD: medication for opioid use disorder.

^e^iRaPID: integrated rapid access to HIV prevention program for people who inject drugs.

^f^PrEP and MOUD educational counseling.

^g^DBS: dried blood spot.

### Strategies To Maximize Recruitment and Retention

Based on our previous research experience [69-72], we are using several strategies to maximize recruitment and minimize attrition rates. Our recruitment strategies include community-based outreach (eg, through flyers and public advertisements), cross-organizational collaborations, and referrals from harm reduction services such as SSPs and mobile clinics. Moreover, the research team routinely meets with the community-based stakeholders (eg, clinical team members, counselors at the MMP, and staff at the SSPs) to ensure proper screening of participants and the smooth function and operation of all study components.

Strategies to promote retention include rapid enrollment, thorough explanation of study requirements, integration of the research program within community-based clinical and nonclinical settings (eg, SSPs, mobile clinics, addiction treatment centers), close monitoring of participants' clinical status, and accessibility of study staff to patients for questions and problems. Participant locator forms, which include name, address, phone number, and family or friend contact, are collected and updated at each visit. Members of the research staff call or message each participant (according to their previously stated preferred contact method) 1 week and 1 day before the date of their visit. We also issue appointment cards at each visit for subsequent ones. Furthermore, participants are compensated based on the time and effort required to complete the assessments based on the market rate, which has also been an essential retention strategy.

### Outcome Measures

The study will assess the feasibility, acceptability, and preliminary efficacy of the iRaPID intervention using a range of instruments administered at various time points throughout the study. The feasibility and acceptability of the program will be evaluated using both quantitative (in-depth interview) and qualitative (one-to-one interview) mechanisms. Data collected include the following: a quantitative assessment of process indicators, standardized acceptability measures collected through quantitative interviews, and measures of fidelity gauged through implementation checklists. Feasibility will be assessed by quantitative process indicators collected by the research staff, including the number of participants screened, recruited, randomized, retained, and adherent to treatment. Acceptability of the intervention will include a 10-item acceptability rating profile and an intervention fidelity assessment. The acceptability profile [73] will be collected through in-depth quantitative interviews at each follow-up point. We will also examine participants' views on acceptability, areas of perceived usefulness of the program, and their confidence in starting PrEP and MOUD. Ultimately, intervention fidelity will be collected using an implementation checklist specific for each part of the program (ie, baseline visit, safety check, follow-up, and clinical visit on day 30), which NPs will complete, documenting the activities covered in each program domain.

We will also estimate the preliminary efficacy of PrEP and MOUD uptake in this pilot RCT of the iRaPID intervention versus TAU (primary outcome). This will be determined by assessing PrEP and MOUD uptake (ie, current use). For those who initiate PrEP, we will also evaluate the time to PrEP initiation, along with adherence and persistence on PrEP, as part of the secondary outcome. Time to PrEP initiation will be calculated by recording the number of days between the first encounter with the NP or health care provider and the day of initiation of PrEP, validated by the pharmacy PrEP pick-up date. We will measure PrEP adherence using the visual analog scale [74] and confirm with dried blood spot testing [75-77]. Persistence on PrEP will be measured by assessing pharmacy data, including both refill (if refilled within 30 days after exhausting PrEP from previous fill or not) and pick-up information. Given the importance of moderating factors on the effect of efficacy, we will measure individual (eg, sociodemographic, sexually transmitted infection, drug use, depression), social (eg, social support), and structural (eg, stigma, physician distrust) factors at each in-depth interview.

### Statistical Analyses

Feasibility will be assessed through the analysis of data collected by the research team, including the number of participants screened and enrolled per month, the proportion of eligible participants who enroll in the study, the treatment-specific proportion of participants completing follow-up visits, rates of adherence to the intervention protocol, and fidelity of implementation measured by the percentage of domains captured by the NPs at each time point. Acceptability analysis will be based on descriptive statistics from the acceptability measures, such as simple means and percentages between treatment arms, and thematic analysis of qualitative data collected in postimplementation focus groups.

Meanwhile, preliminary efficacy analysis will assess the hypothesis that the iRaPID intervention will perform better than TAU over time using a primary outcome (ie, PrEP uptake) and secondary outcomes (ie, time to PrEP initiation, PrEP adherence and persistence). We will first test baseline characteristics for homogeneity between treatment arms using an independent-samples *t* test or Wilcoxon rank sum test (if unequal variances are assessed) for continuous variables and chi-square tests or Fisher exact tests (if any small cell sizes) for categorical variables. This analysis of baseline characteristics will be used to determine significantly different factors to include in the final model for adjustment. To assess our hypotheses relating to preliminary efficacy, we will use a generalized linear mixed model [78] with random subject effects to account for the correlation in repeated measurements that occur within subjects. Treatment assignment, time, and the interaction between time and treatment assignment, including confounders such as sociodemographic characteristics and prior PrEP or MOUD engagement, will be included as covariates. The proportion of participants that initiate PrEP will be estimated and compared using the linear contrast statement in SAS PROC GLIMMIX (SAS Institute). Similar analyses will be conducted for other outcomes, including MOUD uptake, time to PrEP initiation, optimal adherence to PrEP, and PrEP persistence.

### Sample Size

This study aims to test the feasibility and acceptability of the iRaPID program and obtain preliminary estimates of potential effects on HIV prevention outcomes among people who inject drugs. Previous research has shown that pilot studies of 20 to 30 participants per arm are considered adequate to meet proposed study aims similar to this study [79,80]. Our sample size of 50 participants would be ideal for detecting a moderate treatment effect and informing a future RCT. The recruitment of this sample size is feasible based on our formative work.

### Implementation Science Research

As previously mentioned, we are using a type I hybrid implementation trial design, where we will pilot test the NP-led iRaPID program while exploring multilevel implementation factors for future refinement and adoption. As such, we will conduct open-ended interviews with iRaPID participants (n=20) and relevant stakeholders (n=10), including recruitment staff, clinical providers, and administrators at the participating clinic.

We will administer a process measure to assess the overall satisfaction and perceived utility of each intervention component regarding organization-level factors (eg, perceived relevance of intervention components and characteristics to target population needs, the usefulness of the intervention to the organization, and barriers to intervention implementation as designed). These questions will elicit feedback about the potential barriers and facilitators to implementing the iRaPID program—as currently designed—regarding issues ranging from specific intervention components to the more general organizational dynamics (eg., time and resource constraints). The process evaluation will provide a nuanced understanding of the intervention effects, barriers, and facilitators to the intervention implementation and refinement needed to maximize implementation success in a real-world setting. Based on this feedback, the iRaPID program will then be adjusted and adapted for future implementation in various clinical settings.

## Results

Study recruitment began on July 28, 2021, and lasted until August 31, 2022. A total of 174 participants completed screening, yielding 99 eligible participants. The main reasons for ineligibility included not injecting drugs or not having ongoing HIV risk (eg, not engaging in risky sexual or injection drug use practices such as needle sharing). Among eligible participants, 85% (50/99) successfully enrolled in the study and were randomized to a study arm. The remaining eligible participants refused to participate in the study. Enrolled participants had a mean age of 45.98 (SD 8.19) years, 70% (35/50) were male, and 78% (39/50) identified as being heterosexual. They had a diverse portfolio of HIV risk behaviors, as most of them (47/50, 94%) had condomless sex in the past 6 months, and 84% (42/50) injected drugs in the past month. The final study follow-up is expected to be completed by February 2023, and the results will be available by mid-2023.

## Discussion

### Principal Findings and Implications

This ongoing trial aims to test an NP-led integrated HIV prevention program for people who inject drugs that combines same-day coprescribing of PrEP and MOUD. This strategy addresses delayed initiation and low linkage to HIV prevention (PrEP) and substance use (MOUD) care in traditional models. We hypothesize that the iRaPID intervention will be feasible and acceptable among people who inject drugs and will prove to be more efficacious than the standard of care for PrEP and MOUD services. We also anticipate that the results and qualitative feedback from participants and stakeholders will inform the refinement of the iRaPID intervention for a future large-scale RCT.

Same-day access to HIV preventive care may be particularly crucial for people who inject drugs with MOUD as they remain a hard-to-reach group and have significantly higher rates of attrition and poor linkage to care. Since HIV acquisition risk persists during delays, timely PrEP and MOUD initiation are critical in at-risk populations. Individuals with streamlined care initiation may also experience enhanced self-efficacy, encouraging continued engagement. Hence, our results could impact local and international PrEP and MOUD scale-up policies and promote engagement in HIV prevention services.

Given the risk of continued HIV outbreaks amidst the ongoing opioid crisis, scaling up PrEP alongside MOUD for people who inject drugs is crucial for HIV prevention. If successful, subsequent stand-alone tailored programs can be developed to support communities where opioid use is high and HIV outbreaks are likely, thereby promoting a transition to integrative care. Considering the limited access to and scale-up of harm-reduction strategies among people who inject drugs and the existing multilevel barriers to PrEP uptake, effective interventions are urgently needed to fill this critical gap. Given the significant growth in the waivered NP workforce, trained nurses are ideally situated to deliver this integrated care model. The iRaPID program represents a paradigm shift in HIV prevention and may be a particularly crucial strategy for people who inject drugs with OUD, as they remain an easy-to-miss subgroup with high rates of loss to follow-up and poor linkage to care.

### Limitations and Setbacks

The pilot trial has several limitations. Our study is restricted to 1 geographical location (Greater New Haven area, CT), potentially limiting the generalizability of our findings to all people who inject drugs across different geographic settings, particularly areas with fewer or absent SSPs. Further, the prescribing privileges of NPs have geographical disparities of their own, as they only have fully independent practice authority in 29 US commonwealths, states, and territories [81], which undermines nationwide program implementation in areas with physician shortages. Additionally, the study relies on self-reported measures of prior drug use, HIV-related risk behaviors, sexual behaviors, and some measures of primary outcomes (eg, adherence to PrEP using the visual analog scale). Impression management might occur to heighten protocol eligibility, which could potentially lead to the overreporting of risk behaviors during screening and subsequent discrepancies between data collected at different time points. Participants in the pilot trial include both out-of-treatment and in-treatment people who inject drugs (ie, those who were on BMT or MMT at the time of enrollment), which can affect treatment and adherence outcomes, especially as participants who were already on BMT or MMT at the time of enrollment did not undergo induction as compared to their out-of-treatment counterparts.

We also encountered many setbacks regarding participant recruitment due to the COVID-19 pandemic and the recurrent surge of cases in the area. Due to the nature of our program mandating in-person laboratory tests and assessments, ensuing research laboratory closures, and programmatic policies and changes that were developed to ensure research staff safety at the workplace (such as shift schedule changes and remote work) negatively affected recruitment efforts.

### Future Directions

Although shutdown conditions were lifted, insightful strategies implemented by the Addiction Medicine Program at Yale during the COVID-19 pandemic and grasped by the research and clinical staff for future potential use included establishing safe pick-up and delivery of medication and setting up telehealth access for patients. Comparably, the centralization of PrEP dispensing to clinical settings and the in-person nature of its delivery prompted a recent call in the literature for the integration of telehealth to alleviate patient- and provider-level demands [82]. Recent studies, especially in the context of the COVID-19 pandemic, established the feasibility of PrEP and MOUD delivery among people who inject drugs via telehealth [82,83]. Integrating telehealth services within harm reduction settings (eg, SSPs) could not only boost access and address demands imposed on patients [84-86] but also relieve some of the excess pressures imposed on people who inject drugs specifically, including logistical barriers to in-person care and concerns related to stigma. Future directions in implementing the iRaPID program also include expanding access to newly available HIV prevention tools. Preliminary trials have shown that the new Federal Drug Administration–approved, long-acting injectable cabotegravir (given intramuscularly every 8 weeks) is likely to be superior to daily oral tenofovir disoproxil fumarate-emtricitabine (marketed under the name Truvada) in at-risk cisgender men who have sex with men [87]. With the advent of a new mode of administration for PrEP and its ensuing impact on HIV prevention strategies and adherence rates, the iRaPID program will likely have to be restructured to include a long-acting injectable option for participants.

## Appendix

Multimedia Appendix 1 External Peer Review Report from the Population and Public Health Approaches to HIV/AIDS (PPAH) Study Section - Exploratory/Developmental Grants (R21) - National Institute on Drug Abuse (NIDA) (National Institutes of Health, USA).

## Abbreviations

BMT: buprenorphine maintenance therapy
HIPAA: Health Insurance Portability and Accountability Act
iRaPID: integrated rapid access to HIV prevention program for people who inject drugs
MMT: methadone maintenance therapy
MOUD: medication for opioid use disorder
NHSSP: New Haven Syringe Service Program
NP: nurse practitioner
PrEP: pre-exposure prophylaxis
RCT: randomized controlled trial
SSP: syringe services program
TAU: treatment as usual

## Data Availability

All data generated or analyzed up to this point are included in this published article.
