# Supplementary material for: Nurse Practitioner–Led Integrated Rapid Access to HIV Prevention for People Who Inject Drugs (iRaPID): Protocol for a Pilot Randomized Controlled Trial
Source: JMIR Res Protoc. 2022 Oct 11;11(10):e42585. doi: 10.2196/42585 (PMC9597427; doi:10.2196/42585)
Supplement: Multimedia Appendix 1 [file resprot_v11i10e42585_app1.pdf]

SUMMARY STATEMENT

(Privileged Communication)

PROGRAM CONTACT:
[redacted]

Release Date: **03/25/2020**
Revised Date: **03/25/2020**

Application Number: **1 R21 DA051934-01A1**

Principal Investigator

SHRESTHA, ROMAN

Applicant Organization: YALE UNIVERSITY

Review Group: **PPAH**
Population and Public Health Approaches to HIV/AIDS Study Section

Meeting Date: [redacted]
Council: [redacted]
Requested Start: [redacted]

RFA/PA: [redacted]
PCC: [redacted]
Dual IC(s): **NR**

Project Title: **Integrated rapid access to HIV prevention program for people who inject drugs (iRaPID)**
SRG Action: **Impact Score** [redacted] **Percentile:**[redacted]
Next Steps: **Visit https://grants.nih.gov/grants/next_steps.htm**
Human Subjects: **30-Human subjects involved - Certified, no SRG concerns**
Animal Subjects: **10-No live vertebrate animals involved for competing appl.**
Gender: **1A-Both genders, scientifically acceptable**
Minority: **1A-Minorities and non-minorities, scientifically acceptable**
Age: **3A-No children included, scientifically acceptable**

Project Year	Direct Costs Requested	Estimated Total Cost
1	[redacted]	[redacted]
2		
TOTAL	[redacted]	[redacted]

ADMINISTRATIVE BUDGET NOTE: The budget shown is the requested budget and has not been adjusted to reflect any recommendations made by reviewers. If an award is planned, the costs will be calculated by Institute grants management staff based on the recommendations outlined below in the COMMITTEE BUDGET RECOMMENDATIONS section.

REVISION NOTE

1R21DA051934-01A1 Shrestha, Roman

RESUME AND SUMMARY OF DISCUSSION: The applicant proposes developing and pilot testing a nurse delivered same day, rapid access to PrEP and opiate antagonist therapy (OAT) to persons who inject drugs (PWID) labeled iRaPID. The premise for this application is very strong as data from this group supported by the extant literature show that following screening of PWID on OAT to determine eligibility for PrEP, a process that can take up to three weeks, a large number drop out and fail to show up for PrEP initiation. The vulnerability of this population to HCV and HIV underscores the significance of this focus and the need for iRaPID. This outstanding team of investigators has contributed substantively to knowledge on substance abuse and there is no doubt this project will shed much needed light on the efficacy of providing rapid access to PrEP and OAT to this population. The engagement of Advance Practice Nurses (APN) in delivering this intervention is innovative and sensitive to the clinical needs of PWID. This study which will be carried out within an implementation science framework will test the acceptability and feasibility of iRaPID for PWID and healthcare providers and will compare the preliminary efficacy of PrEP and OAT uptake in iRaPID vs treatment as usual among PWID without HIV. This resubmission was very responsive to previous critiques and the applicants have clarified the various issues raised and thus the application has been much improved. While the application was said to be ambitious, not only was it felt that this is the right application mechanism to explore this concept, but also, the experience of the applicants in conducting projects of this scope raised confidence that they can undoubtedly get the job done. There were some differences of opinion regarding some weaknesses in the application, including concern about APN burden by expanding their workload; a lack of integration between the qualitative and quantitative components of the application, and remaining concerns about the feasibility of the project. Nevertheless, these were not compelling to the vast majority of the committee who thought this application very meritorious and assess its potential impact as very high.

DESCRIPTION (provided by applicant): The HIV epidemic among people who inject drugs (PWID) has been on the decline, but amidst a burgeoning opioid epidemic, communities are now increasingly vulnerable to HIV transmission. Recent HIV outbreaks linked to drug injection has introduced HIV into PWID networks and thus, potentially reverse decades of HIV prevention successes. While opioid agonist therapy (OAT) and syringe services programs (SSPs) reduce HIV transmission, access to and utilization of such programs are unavailable or with limited availability; sexual and injection-related HIV risks persist in many PWID. The integration of pre-exposure prophylaxis (PrEP) into existing evidence-based programs (e.g., OAT, SSPs) has been presented as an opportunity to strengthen HIV prevention efforts in PWID. Uptake, however, remains stubbornly low in PWID despite them being ideal candidates and interested in starting PrEP. Data from our ongoing PrEP adherence trial in PWID show high rates of attrition (43.7%) between the initial PrEP eligibility screening visit and PrEP initiation (usually 1-3 weeks). Further, qualitative interviews indicate preferences for PrEP delivery that would decrease waiting times or repeated visits altogether PrEP prescription. These early findings, supported by others, guide the need for rapid PrEP initiation integrated within an existing harm reduction services that reduce or eliminates patient, clinician, and structural barriers. Results from recent pilot studies have shown early acceptability, feasibility, and safety of rapid (same-day) PrEP initiation in men who have sex with men (MSM) and transgender women (TGW), but none of them include PWID, a group with extraordinary need in the current opioid crisis. Rapid PrEP initiation may be particularly important for PWID as they are more likely to be lost before treatment initiation. To fully optimize HIV prevention, PrEP care should be combined with OAT. Combining OAT with ART evolved from physicians who would withhold antiretroviral therapy (ART) from PWID if they were using drugs; if patients were OAT, ART prescription increased. Given the findings that advanced practice nurses (APNs) are more likely to inquire in a patient-centered manner about their drug use and provide more supportive counseling, a

new differentiated care model of combined, same-day PrEP/OAT for PWID is well-suited to start with APNs. We, therefore, propose to develop and pilot test this model within an implementation science framework. The specific aims are to: 1) Aim 1: examine feasibility and acceptability among PWID and clinical stakeholders for an adapted APN-delivered, rapid HIV prevention program for PWID (iRaPID) that integrates same-day PrEP and OAT; and 2) Aim 2: estimate the preliminary efficacy of PrEP and OAT uptake in a pilot randomized controlled trial of the iRaPID vs. treatment as usual strategy in PWID without HIV. Together, these aims will address a wide gap in HIV prevention by addressing multilevel barriers to dispensing same-day combination prevention. Elements learned from a successful same-day PrEP/OAT model for PWID can guide future scale-up models that incorporate both APNs and physicians in urban and non-urban settings where resources are limited.

PUBLIC HEALTH RELEVANCE: The opioid epidemic in the United States, especially the rise in injection drug use, necessitates the need for novel strategies to reduce the risk of HIV infection in people who inject drugs (PWID). The proposed research aims to jump-start the HIV prevention cascade by developing and pilot-testing a nurse-delivered, integrated rapid access to HIV prevention program for PWID (iRaPID) program that incorporates same-day access to pre-exposure prophylaxis (PrEP) and opiate agonist therapy (OAT). Findings will inform the development of innovative and tailored primary HIV prevention strategy to address co-occurring sexual and drug risk behaviors and to enhance the HIV prevention gap in PWID amid the ongoing opioid crisis.

CRITIQUE 1

Significance: 2
Investigator(s): 2
Innovation: 2
Approach: 4
Environment: 2

Overall Impact: This proposed study is to assess feasibility, acceptability and preliminary efficacy of an adapted APN-delivered, rapid HIV prevention program called iRaPID that integrates same-day PrEP and opioid agonist therapy (OAT) among people who inject drugs (PWID). The project has novel components, like the rapid PrEP + OAT starts at the same time to decrease attrition from screening to initiation among PWID and the use of advanced practice nurses for PrEP and OAT delivery to expand beyond physician prescription and delivery. The team is very well organized with support from Gilead to provide PrEP, if needed, and strong community collaborations ensuring the project will be feasible with high potential for success. This resubmission was responsive to the prior review but concerns around feasibility of this project in the given time frame remain high. There are some components of the proposal that could have been provided in more detail like the PrEP eligibility and plans for retention, which are very important in PrEP delivery and would be of considerable importance for this underserved population. Despite some remaining limitations, the project is innovative, high risk/reward, and meets a significant gap in the rollout of PrEP, which has almost entirely passed over people who inject drugs and could highly benefit from this important HIV prevention tool. Due to these reasons, enthusiasm for this proposal is high.

1. Significance:

Strengths

- The scale up of PrEP among PWID is highly inadequate, despite the urgent need, and this proposal seeks to provide an immediate attempt to address this large gap in the PrEP scale up efforts.

- Rapid PrEP delivery with OAT has the ancillary and potentially crucial benefit of preventing attrition in PrEP as PWID may be have increased motivation to maintain OUD treatment.

Weaknesses

- It is not clear that acceptability data are needed with PWID as there is evidence of acceptability of OAT and PrEP, and data cited by the team showing attrition from screening to PrEP initiation among PWID.
- Justification for focusing only on APNs seems to be a limiting factor to the impact of this proposal. Inclusion of physicians seems important and necessary given the current state of APN prescribing limitations in some states.
- Justification for OAT and PrEP integration needs more detail. If OAT and PrEP are both a problem, there may be a negative synergy when both are offered.

2. Investigator(s):

Strengths

- The team of investigators at Yale has a long and successful record of observational research and intervention research that are needed with PWID and in the existing community collaborations to make this project feasible and likely to be successful.

Weaknesses

- None Noted by Reviewer

3. Innovation:

Strengths

- Focus on rapid OAT and PrEP among PWID is novel and builds an efficiency that can optimize benefits for community members.

Weaknesses

- None Noted by Reviewer

4. Approach:

Strengths

- Availability of PrEP access via the new Set, Ready, PrEP is an important historical improvement that will better facilitate PrEP access.
- Same day starts for PrEP is important and the right approach to increasing PrEP access.
- Strong plan for clinic visit checklist and important to fidelity checks.
- Established partnership with Gilead is important to ensure rapid starts for PWID interested and eligible to take PrEP.
- Seems like an implementation science approach where all participants receive intervention is appropriate given the team's data on attrition b/t screening and initiation of PrEP in prior research

Weaknesses

- If aim one does not show feasibility and acceptability, then the team will not have evidence to support moving forward
- An important component of the formative work will be to measure, with some level of specificity, additional hours of work on APNs given follow up, and lengthier enrollment visit, etc. to determine feasibility. The additional workload may provide the most important barrier to successful implementation.
- NGTs would be more useful at the end of the study given that the intervention will be tested regardless and the approach is new and clinicians and participants may not be able to anticipate challenges they will face in implementation.
- PrEP eligibility screening is unclear. Are all people who do not have contraindications for PrEP eligible?
- Will this research be biased by PrEP study currently underway with PWID?
- Burden of after work hours participation of providers in NGT seems high. Individual interviews would be a better fit.
- The NGT questions seem straight forward and it is not clear that 5 groups are needed to address identifying barriers to PrEP and OAT delivery and integration.
- Methods for retention are not particularly robust. Though prescribing OAT may help, assisting PWID with PrEP persistence, not just initiation, is essential to reaping the benefit of PrEP.
- The extraordinary use of acronyms, including those not spelled out previously (e.g. TAU) makes for a hard read of the proposal. The writing is also a bit challenging to read and could benefit from some additional proofing.

5. Environment:

Strengths

- The letters of support demonstrate the strong community buy in of the community-based organizations.
- The Yale University School of Medicine and Community Health Care Van (CHCV) and the New Haven Syringe Services Program (NHSSP) are long standing research community collaborations and a strong setting for the research.

Weaknesses

- None Noted by Reviewer

Study Timeline:

Strengths

- The timeline is better articulated lessening concern about the feasibility of the timeline for the qualitative Phase I activities.

Weaknesses

- Phase II activities still seem ambitious. The team will have to recruit and randomize 90 participants into a 6 months intervention in a 2 year total project along with the qualitative phase and training of APNs on this newly developed protocol.

Protections for Human Subjects:

Acceptable Risks and/or Adequate Protections

Data and Safety Monitoring Plan (Applicable for Clinical Trials Only):

Acceptable

Inclusion Plans:

- Sex/Gender: Distribution justified scientifically
- Race/Ethnicity: Distribution justified scientifically
- For NIH-Defined Phase III trials, Plans for valid design and analysis: Not applicable
- Inclusion/Exclusion Based on Age: Distribution justified scientifically

Vertebrate Animals:

Not Applicable (No Vertebrate Animals)

Biohazards:

Not Applicable (No Biohazards)

Resubmission:

- The investigators were responsive to comments from reviewers; however, the response to #3 that the project was ambitious given the time, budget and amount of work proposed to complete, with the reviewer's being asked to believe the project is feasible based on the successes of Co-Investigators' work on prior project and explanation that nominal group process findings are easily retrievable without in-depth analysis. The project remains ambitious but is a good fit for the R21 mechanism.

Resource Sharing Plans:

Acceptable

Authentication of Key Biological and/or Chemical Resources:

Not Applicable (No Relevant Resources)

Budget and Period of Support:

Recommend as Requested:

- Biomarker costs not included

CRITIQUE 2

Significance: 5
Investigator(s): 3

Innovation: 2
Approach: 4
Environment: 1

Overall Impact: The team proposes to develop and pilot test an intervention (termed iRAPID) for persons who inject drugs (PWID) that is designed to augment PrEP uptake by incorporating same day access to opiate agonist therapy (OAT) and delivered by advanced practice nurses (APNs). The team proposes a highly innovative application with an exceptionally experienced team. However, the focus on PWID who are on OAT for PrEP is not posited to address the population most at risk for HIV incidence (young PWID who are not on OAT), dampening the significance of the study. Additional moderate weaknesses in the approach, including an overly ambitious timeline, lack of integration of the quantitative and qualitative data, and questionable use of the implementation framework to simultaneously evaluate two interventions (OAT, PrEP) also diminish enthusiasm for the overall application.

1. Significance:

Strengths

- HIV infections among all PWID continues to increase with the expansion of the opioid epidemic.
- Need to address strategies to increase uptake of both OAT and PrEP.

Weaknesses

- The co-management of HIV risk with PrEP and OAT among PWID with active drug use requires consideration of many factors in addition to access to medication and facilitation of linkage to care.
- Both OAT and PrEP interventions are highly stigmatized and suffer from low adherences on their own. Thus, trying to address both drug use and HIV risk with OAT and PrEP at the same time might compound these issues among PWID.
- More impact on HIV incidence from any intervention is posited for PWID at highest risk for HIV, i.e., those not connected to any services, including OAT and harm reduction services. Focusing on PWID who have already decided to address their drug use with OAT—i.e., those who have already reduced their risk for HIV by reducing the need to share syringes may not have added value to reducing HIV incidence in this population.
- The single age group of PWID with the highest incidence are 13-34 year old (45% of all cases reported by the CDC in 2018. This group is also most likely to be in the early years of their injection drug use and not linked to OAT. The proposed study focuses on all PWID over age 18, which does not address the population most in need of these interventions.

2. Investigator(s):

Strengths

- The junior faculty has adequate experience to successfully carry-out this project given the support of seasoned investigators on the team.

Weaknesses

- PI effort allocated to the project (20%) seems low given the extensive tasks associated with his role.

3. Innovation:

Strengths

- Addressing HIV risk (PrEP) and drug use management (OAT) simultaneously in a single intervention.

Weaknesses

- None Noted by Reviewer

4. Approach:

Strengths

- RCT design is likely to produce robust results.

Weaknesses

- The use of a hybrid implementation model may not be appropriate for evaluation of two evidence-based interventions simultaneously (i.e., both OAT and PrEP uptake).
- The integration of the qualitative and quantitative components is not described.
- Literature presented to support safety of PrEP and OAT is inadequate (e.g., reports on safety of PrEP among HBV-infected with no evidence of OAT use, reference # 183)
- Timeline for proposed study is highly infeasible—as there is no room for any potential delays of any kind.

5. Environment:

Strengths

- Resources and environment at both performance sites is adequate for the completion of the project.

Weaknesses

- None Noted by Reviewer

Study Timeline:

Strengths

- None Noted by Reviewer

Weaknesses

- The sequential (linear) design with a tight timeline raises concern of the impact of potential delays year 1; the activities proposed for the first 6 months alone is ambitious for a full year of a clinical trial.

Protections for Human Subjects:

Unacceptable Risks and/or Inadequate Protections

- The only study cited for addressing safety of PrEP and OAT is reference # 183. This reviewer read this study and there is no data on OAT in the population being studied (those with active

HBV infection). Additional studies on the parent project (iPrEx study) were reviewed and, again, no evidence of OAT being evaluated within the context of PrEP.

Data and Safety Monitoring Plan (Applicable for Clinical Trials Only):
Acceptable

Inclusion Plans:

- Sex/Gender: Distribution justified scientifically
- Race/Ethnicity: Distribution justified scientifically
- For NIH-Defined Phase III trials, Plans for valid design and analysis: Not applicable
- Inclusion/Exclusion Based on Age: Distribution justified scientifically
- Appropriate.

Vertebrate Animals:
Not Applicable (No Vertebrate Animals)

Biohazards:
Not Applicable (No Biohazards)

Resubmission:

- Many of the concerns were addressed; however, two concerns were not adequately addressed. These include 1) timeline to complete the project (too ambitious)--the PI cited evidence of prior experience unrelated to the issue, i.e., longitudinal studies with more time to adjust and respond to year 1 delays than an R21; 2) the safety of initiating PrEP and OAT; the PI cites evidence in support of this that were irrelevant, i.e., concurrent OAT and PrEP use was not evidence in the two supporting citations (#183: safety of PrEP among HBV-infected patients and #184: reversal of glomerular renal function among those on PrEP only)

Resource Sharing Plans:
Not Applicable (No Relevant Resources)

Authentication of Key Biological and/or Chemical Resources:
Not Applicable (No Relevant Resources)

Budget and Period of Support:
Recommended budget modifications or possible overlap identified:

- PI effort seem low; recommend adjusting.

CRITIQUE 3

Significance: 2
Investigator(s): 1
Innovation: 3
Approach: 2
Environment: 1

Overall Impact: This R21 proposes a RCT to test a rapid intervention delivered by Advanced Practitioner Nurses that includes same day PrEP and Opioid Antagonist therapy to people who inject drugs. Given the recent increase of HIV among people who inject drugs, this intervention could have a major impact on public health.

This is a novel intervention, combining PrEP and OAT for people who inject drugs. The investigative team is very strong. They make a compelling case that they can conduct this ambitious project within the proposed timeline, based on their experience with previous projects. This project is also relevant because it is tracking the trend of making the scope of APNs' practice more broad in more states. This R21 application is highly responsive to the Program Announcement and to the weaknesses identified in the previous review. Overall, impact is judged to be in the high range.

1. Significance:

Strengths

- The need to address HIV infection among people who inject drugs remains a priority.
- The need to reduce barriers to PrEP in this population is high.
- If carried out successfully, this project may lead to significant and lasting changes in this area of health care delivery and impact HIV rates among people who inject drugs.

Weaknesses

- None Noted by Reviewer

2. Investigator(s):

Strengths

- The investigative team is extremely strong, with expertise in all relevant areas of the project.
- The addition of Drs. Altice and Vlahov give great confidence that the project with people who inject drugs will be carried out successfully.

Weaknesses

- None Noted by Reviewer

3. Innovation:

Strengths

- This would be the first intervention to combine same day PreP and opioid agonist therapy for people who inject drugs to reduce risk of HIV.
- The use of APNs to deliver the intervention is also novel.

Weaknesses

- Other methods are not particularly novel, but that does not diminish enthusiasm.

4. Approach:

Strengths

- The application is highly responsive to the few weaknesses raised in the previous review.
- The scope of the proposed activities seems appropriate for an R21 testing a new idea.
- Integration of the trial within the CFIR framework is strong.

Weaknesses

- Minor; The widespread ability of APNs to deliver the intervention remains a possible limitation. However, the if legislation does in fact change, expanding APN practice scope, making this more possible, it will be good to know if this intervention is feasible and possibly effective.

5. Environment:

Strengths

- The academic environment at Yale is exceptional and will support the proposed project.

Weaknesses

- None Noted by Reviewer

Study Timeline:

Strengths

- The timeline is ambitious for carrying out the two aims. However, the team has a history of successfully carrying out projects that are similarly rapid.

Weaknesses

- None Noted by Reviewer

Protections for Human Subjects:

Acceptable Risks and/or Adequate Protections

- The application shows experience working with PWID and the human subjects research issues relevant to this group. The

Data and Safety Monitoring Plan (Applicable for Clinical Trials Only):

Acceptable

Inclusion Plans:

- Sex/Gender: Distribution justified scientifically
- Race/Ethnicity: Distribution justified scientifically
- For NIH-Defined Phase III trials, Plans for valid design and analysis: Not applicable
- Inclusion/Exclusion Based on Age: Distribution not justified scientifically

- Children and adults age 18 and older. No restriction on gender or ethnicity. Participants in New Haven, CT for this pilot study.

Vertebrate Animals:

Not Applicable (No Vertebrate Animals)

Biohazards:

Not Applicable (No Biohazards)

Resubmission:

- The resubmission is highly responsive to previous critiques.

Resource Sharing Plans:

Not Applicable (No Relevant Resources)

Authentication of Key Biological and/or Chemical Resources:

Not Applicable (No Relevant Resources)

Budget and Period of Support:

Recommend as Requested

Recommended budget modifications or possible overlap identified:

THE FOLLOWING SECTIONS WERE PREPARED BY THE SCIENTIFIC REVIEW OFFICER TO SUMMARIZE THE OUTCOME OF DISCUSSIONS OF THE REVIEW COMMITTEE, OR REVIEWERS' WRITTEN CRITIQUES, ON THE FOLLOWING ISSUES:

PROTECTION OF HUMAN SUBJECTS: ACCEPTABLE

INCLUSION OF WOMEN PLAN: ACCEPTABLE

INCLUSION OF MINORITIES PLAN: ACCEPTABLE

INCLUSION ACROSS THE LIFESPAN: ACCEPTABLE

COMMITTEE BUDGET RECOMMENDATIONS: The budget was recommended as requested.

REVISION NOTE: This summary statement was revised to update comments made by reviewer 2

Footnotes for 1 R21 DA051934-01A1; PI Name: Shrestha, Roman

+ Derived from the range of percentile values calculated for the study section that reviewed this application.

NIH has modified its policy regarding the receipt of resubmissions (amended applications).See Guide Notice NOT-OD-18-197 at https://grants.nih.gov/grants/guide/notice-files/NOT-OD-18-197.html. The impact/priority score is calculated after discussion of an application by averaging the overall scores (1-9) given by all voting reviewers on the committee and multiplying by 10. The criterion scores are submitted prior to the meeting by the individual reviewers assigned to an application, and are not discussed specifically at the review meeting or calculated into the overall impact score. Some applications also receive a percentile ranking. For details on the review process, see http://grants.nih.gov/grants/peer_review_process.htm#scoring.
